# Pupil response hazard rates predict perceived gaze durations

**DOI:** 10.1038/s41598-017-04249-9

**Published:** 2017-06-21

**Authors:** Nicola Binetti, Charlotte Harrison, Isabelle Mareschal, Alan Johnston

**Affiliations:** 1 Department of Experimental Psychology, University College London, London, UK; 2 School of Biological and Chemical Sciences, Psychology, Queen Mary University of London, London, UK; 3 CoMPLEX, University College London, London, UK; 4 School of Psychology, University of Nottingham, Nottingham, UK

## Abstract

We investigated the mechanisms for evaluating perceived gaze-shift duration. Timing relies on the accumulation of endogenous physiological signals. Here we focused on arousal, measured through pupil dilation, as a candidate timing signal. Participants timed gaze-shifts performed by face stimuli in a Standard/Probe comparison task. Pupil responses were binned according to “Longer/Shorter” judgements in trials where Standard and Probe were identical. This ensured that pupil responses reflected endogenous arousal fluctuations opposed to differences in stimulus content. We found that pupil hazard rates predicted the classification of sub-second intervals (steeper dilation = “Longer” classifications). This shows that the accumulation of endogenous arousal signals informs gaze-shift timing judgements. We also found that participants relied exclusively on the 2^nd^ stimulus to perform the classification, providing insights into timing strategies under conditions of maximum uncertainty. We observed no dissociation in pupil responses when timing equivalent neutral spatial displacements, indicating that a stimulus-dependent timer exploits arousal to time gaze-shifts.

## Introduction

Evidence from both primates and humans has shown that gaze direction processing is embedded in dedicated neuronal circuitry. Electrophysiological recordings in the anterior superior temporal sulcus (STS) of macaque monkeys have shown anatomically segregated cell populations responsive to direct and averted gaze^1, 2^. Human functional imaging studies have documented posterior STS activation in gaze processing^3, 4^ and distinct neural systems in the anterior STS and inferior temporal lobule for the encoding of rightward and leftward gaze shifts^5^. In particular, direct gaze is known to modulate cognition and attention^6^ and evoke activity in specialized brain areas as compared to averted gaze^7, 8^. However, any evaluation of gaze processing is incomplete without an understanding of how we estimate the duration of gaze behaviours. Recently we identified in a 500 participant sample the duration that constitutes a “normal” amount of eye contact, and related individual preferences in gaze duration to changes in pupil dilation (an index of physiological arousal)^9^. We found that on average people favour a 3 second period of eye contact, which is largely independent of demographic and personality variables. We also found that rate of pupil dilation is a predictor of preferred eye contact duration: participants that preferred longer periods of direct gaze exhibited faster rates of pupil increase. In the present study we aimed at investigating the mechanisms through which people evaluate the duration of other’s gaze behaviours.

Timing is achieved throughout a distributed network of cortical and subcortical structures, including the supplementary motor area, the cerebellum, the basal ganglia and the thalamus^10^ and is revealed by endogenous signals which reflect subjective duration^11–13^. Cortical arousal has been frequently linked to time: arousing stimuli lead to overestimated durations^14^. This has typically been framed as arousal increasing the clock rate of a universal timekeeping mechanism^15^. The limitation of these approaches is that one cannot determine whether the effects on time perception are driven by variations in arousal, variations in stimulus content (different stimuli are used to elicit different arousal responses), or some combination of both.

Here we consider arousal, measured through pupil dilation, as a candidate for an endogenous timing signal that may contribute to gaze-shift timing judgements. In the first experiment participants judged the duration of gaze shifts performed by avatar stimuli within a standard binary choice psychophysical task. In order to disentangle differences in arousal from differences in sensory content, we directly evaluated how differences in arousal contribute to differences in perceived duration for identical stimuli. We found that sub-second gaze shift stimuli classified as longer were accompanied by steeper increases in pupil diameter, thus showing that an accumulation of endogenous arousal signals, indexed by an evoked pupil response, drove duration classifications. This is consistent with an accumulation of timing evidence used by the brain to anticipate when an event is likely to occur, i.e. the Hazard function. In a second experiment we evaluated whether the pupillary responses were stimulus dependent or generic to the structure of the task by having participants judge the duration of equivalent Gabor carrier shifts. We found that pupil hazard rates did not predict participant duration classifications. These results show that a stimulus dependent sub-second timing system exploits endogenous arousal signals to estimate gaze shift duration.

## Results

**Figure 1:**
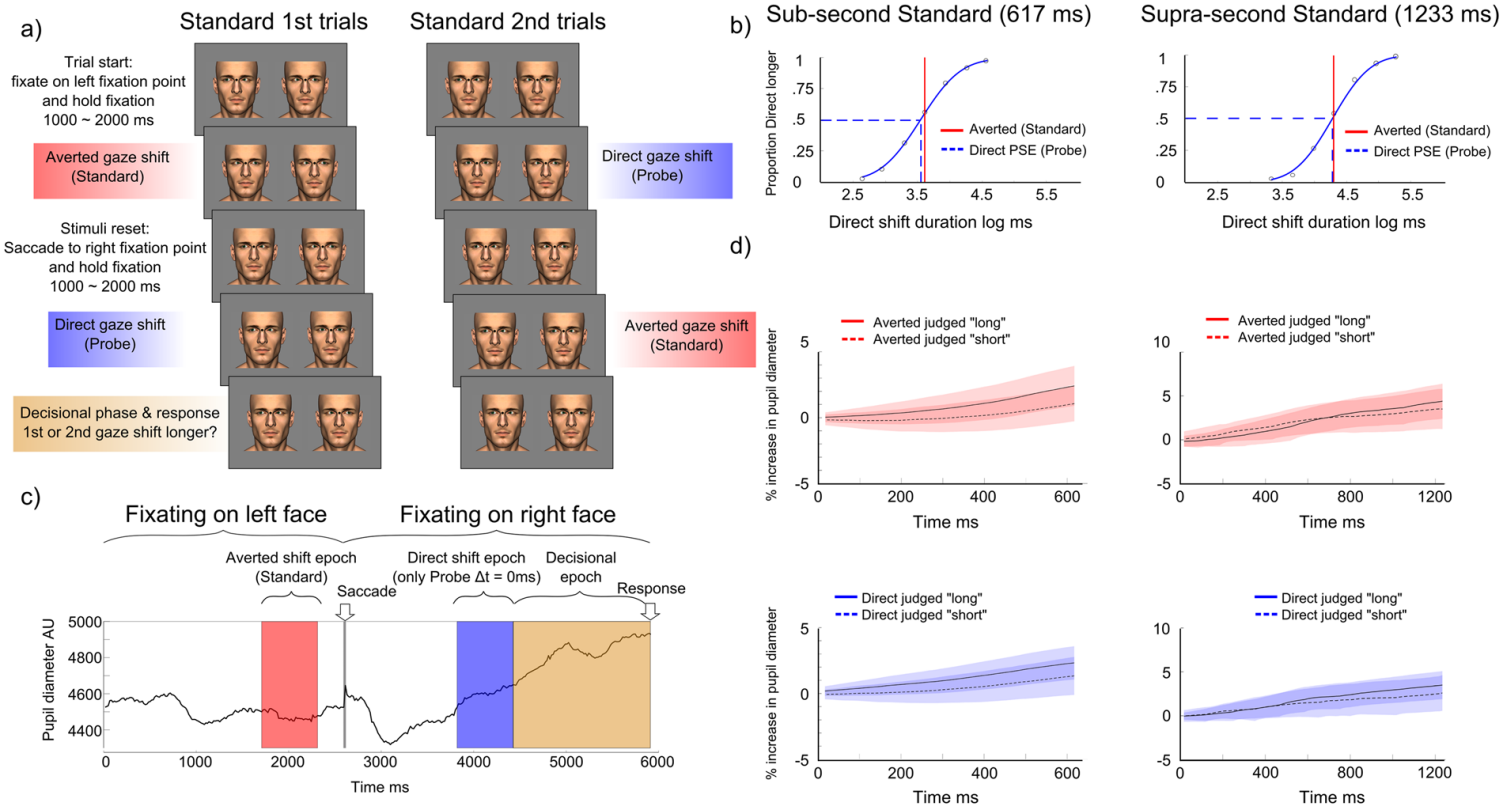
(**a**) Gaze shifts were performed by the left avatar first, and the right avatar second. Participants saccade from the left to the right fixation point when the left avatar has reset to its starting position following its gaze shift. The order of direct and averted shifts was counterbalanced across trials, so on half the trials the left avatar performed an averted shift and then the right avatar performed a direct shift (Standard 1^st^ trials), while in the remaining half the left avatar performed a direct shift and then the right avatar performed an averted shift (Standard 2^nd^ trials). (**b**) Group average psychometric functions for sub and supra-second standards. The Point of Subjective Equality (PSE – blue dotted line) indicates the duration required for a Direct gaze shift to appear equally long to an Averted gaze shift. The red solid line represents the standard duration. (**c**) Pupil signal segmentation into Direct & Averted *duration encoding* epochs and the ensuing *decisional* epoch in a “Standard 1^st^” trial. (**d**) Pupil signal *response type* comparison during the direct and averted *duration encoding* epochs. We directly compared pupil responses across identical stimuli that lead to opposite duration classifications. Shaded error plots show averaged increase in pupil diameter across time during gaze shifts of identical physical duration classified as “long” or classified as “short”. Avatar stimuli created with Poser 9 software (SmithMicro Software).

In Experiment 1, participants compared the duration of Averted (Standard) and Direct (Probe) gaze shifts sequentially performed by two face stimuli in a Standard/Probe binary choice task (Fig. 1a). Group average psychometric functions for sub & supra-second standards are displayed in Fig. 1b, which shows the Point of Subjective Equality for Direct shifts (PSE), relative to the Averted standards, in sub and supra-second blocks. Within-subject t-test comparisons revealed no overall differences in the apparent durations of Direct shifts (PSEs) and Averted shift standards (sub-second Standard: t(9) = −1.88, p = 0.26; supra-second Standard: t(9) = −0.1, p = 0.34).

**Figure 2:**
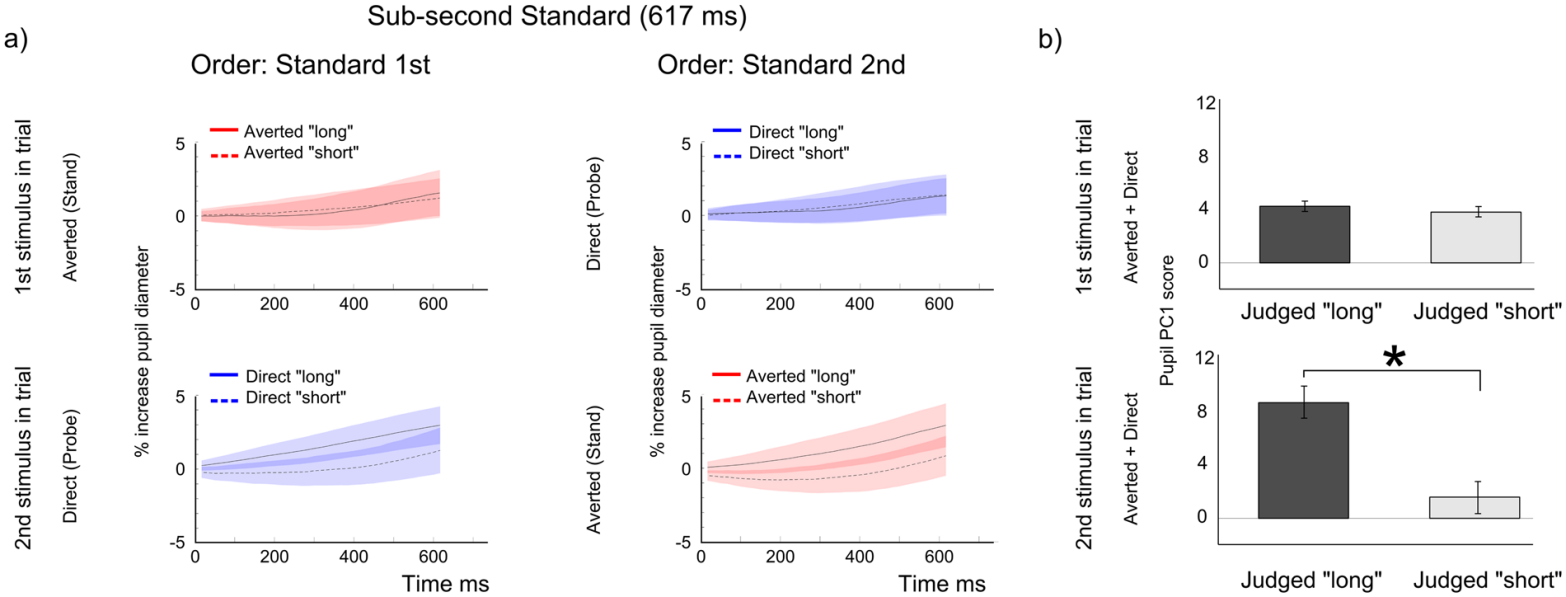
(**a**) Averaged pupil responses during the direct and averted *duration encoding* epochs as a function of duration judgement (longer or shorter) and stimulus order (Standard 1^st^: averted 1^st^ – direct 2^nd^, or, Standard 2^nd^: direct 1^st^ – averted 2^nd^). (**b**) Pupil PC1 score as a function of duration judgement (longer or shorter). Error bars depict the Standard Error of the Mean (SEM).

Figure 1c illustrates epochs of interest within the time course of a single trial. Averaged pupil signals were produced by binning pupil responses based on “Longer/Shorter” duration judgements in trials where the durations of the Standard (averted shift) and Probe (direct shift) were identical (Fig. 1d). Averaged pupil signals during the *duration encoding* epochs (marked as red and blue) showed a relatively linear rate of pupil dilation from the onset of the timed interval. We found that pupil responses associated with ‘longer’ and ‘shorter’ classifications of sub-second stimuli differ in terms of rate of pupil dilation (i.e. the slope of the functions). We tested whether there was a difference in pupil response during the *duration encoding* epochs by submitting PC1 scores, derived from a principal components analysis of the pupil diameter time series (see Supplementary Information), which summarize the rate of pupil dilation (greater PC1 score – greater increase in pupil diameter over time), to a 2 × 2 × 2 Repeated Measures ANOVA with factors Stimulus Order (Standard 1^st^ Vs Standard 2^nd^) × Gaze Type (Direct Vs Averted shift) × Duration Judgement (judged Longer Vs Shorter). This analysis revealed a main effect of Duration Judgement (F(1, 9) = 11.96, p = 0.007): stimuli judged ‘Longer’ were associated with steeper slopes with respect to stimuli judged ‘Shorter’. Thus, when timing sub-second direct and averted stimuli, differences in pupil signal reflect differences in perceived gaze shift duration. We also observed a significant Stimulus Order × Gaze Type × Duration Judgement interaction (F(1, 9) = 7.62, p = 0.02). We explored this interaction by comparing Direct & Averted duration judgement comparisons for each stimulus order condition separately (Fig. 2a). A 2 × 2 ANOVA, with factors Gaze Type and Duration Judgement was conducted on PC1 scores associated with the encoding of the 1^st^ stimulus in the trial (Fig. 2b, top row). No significant main effects and no significant interaction was observed. We ran an equivalent 2 × 2 ANOVA on PC1 scores associated with the encoding of the 2^nd^ stimulus in the trial, which only revealed a main effect of Duration judgement (F(1, 9) = 10.37, p = 0.01) (Fig. 2b, bottom row). This indicates that a significant difference in pupil dilation rates for longer and shorter judgements only occurred when the stimulus (either a direct or averted shift) occurred in the second phase of the trial.

**Figure 3:**
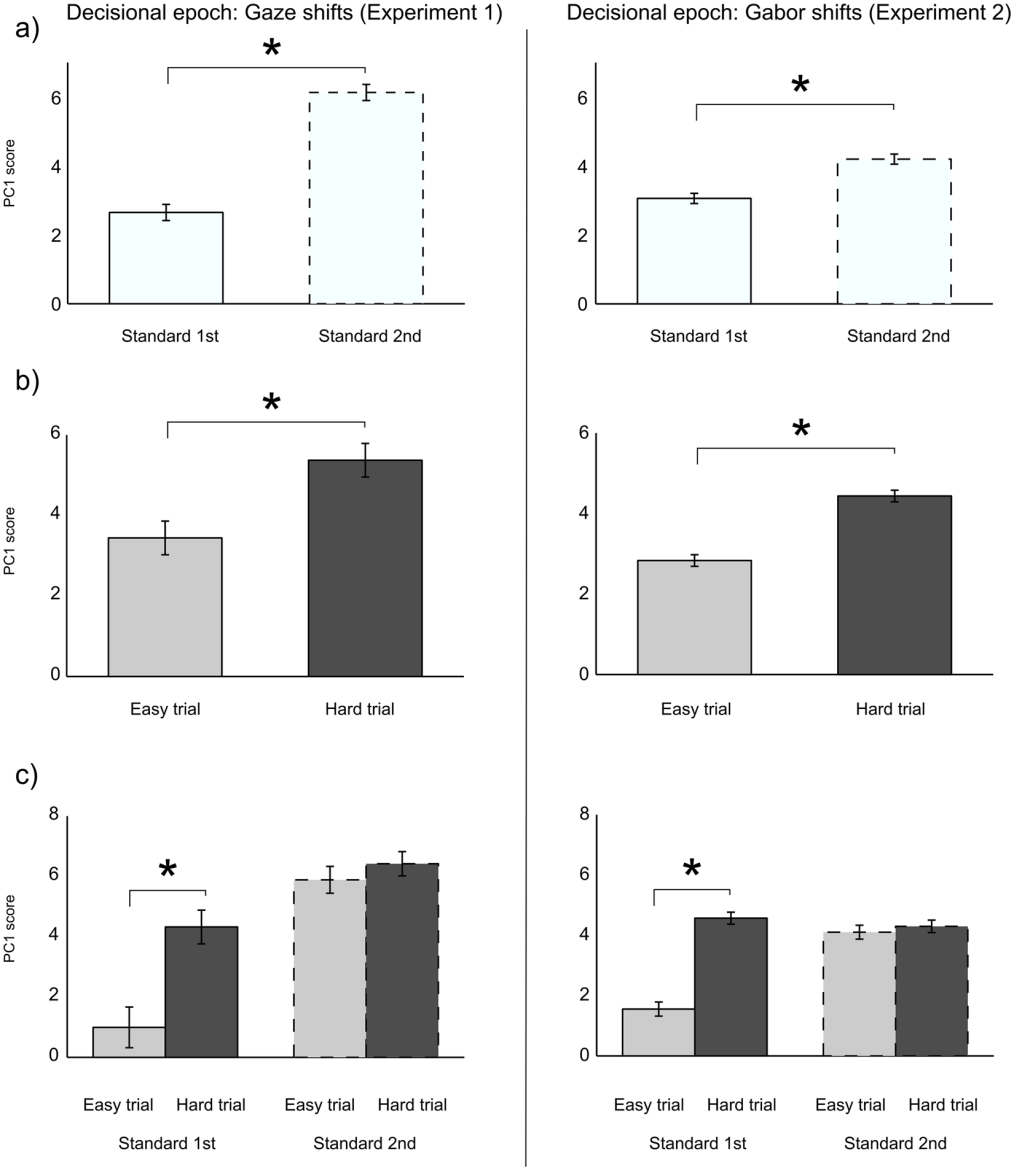
Decisional epoch pupil responses associated with the duration classification of Gaze shift stimuli (Experiment 1, left column) and Gabor shift stimuli (Experiment 2, right column). (**a**) Rate of pupil increase during the decisional epoch (PC1 score) as a function of stimulus order in trial: trials in which the Standard follows the Probe (Standard 2^nd^) are characterized by faster increases in pupil diameter with respect to trials in which the Standard precedes the Probe (Standard 1^st^), (**b**) PC1 score during the decisional epoch as a function of trial difficulty: hard trials (where standard and probe have same duration, thus being harder to classify) exhibit faster increases in pupil diameter with respect to easy trials (where Standard and Probe have noticeably different durations), (**c**) PC1 score during the decisional epoch as a function of trial difficulty and stimulus order: difference in pupil response between hard and easy classifications is only observed in trials in which Standard occurs 1^st^ (i.e. decisional epoch occurs immediately after the offset of the Probe).

We also submitted PC1 scores associated with the *decision epoch* (following the offset of the 2nd stimulus, Fig. 1a,c) to a 2 × 2 repeated measures ANOVA, with factors Stimulus Order (Standard 1st Vs Standard 2nd) and Trial Difficulty (Hard Vs Easy). Hard trials consist of Standard and Probe stimuli of equal duration, while easy trials contain noticeably short or long Probes, which are easier to classify. We found a main effect of Stimulus Order (F(1, 9) = 55.39, p = 4^e−05^): pupil responses during the decisional phase are steeper in trials where the Standard (averted) occurs 2^nd^ (Fig. 3a, left panel). We also found a main effect of Trial Difficulty (F(1, 9) = 5.32, p = 0.046), revealing that harder trials are characterized by steeper pupil dilation responses (Fig. 3b, left panel), and a significant Stimulus Order × Trial Difficulty interaction (F(1, 9) = 9.44, p = 0.01), indicating the dissociation in pupil dilation response between hard and easy trials is only observable when the decision phase directly follows the Probe (direct shift) (Fig. 3c, left panel).

Equivalent analyses were run on data from the supra-second blocks. No significant main effects or interactions were observed during the *duration encoding* epochs or during the *decision making* epoch.

In Experiment 2 participants timed two sequential Gabor phase-shifts (Supplementary Information, Figure S1). This was carried out to assess whether the pupillary responses uncovered in Experiment 1 were stimulus-dependent or generic to the structure of the task. *A* 2 × 2 Repeated Measures ANOVA with factors Stimulus Order (Standard 1st Vs Standard 2nd) × Duration Judgement (judged Longer Vs Shorter) revealed no significant differences in pupil response during the *duration encoding* epochs. Thus, we did not find a dissociation in pupil responses as a function of the duration classifications, independently of stimulus order, with this stimulus type (Supplementary Information, Figure S2).

A 2 × 2 Repeated Measures ANOVA with factors Stimulus Order (Standard 1st Vs Standard 2nd) × Trial Difficulty (Hard Vs Easy) run on pupil PC1 scores during the *decision epoch* revealed consistent with the gaze shift dataset, a main effect of Stimulus order (F(1, 9) = 14.9, p = 0.004), indicating that pupil dilation responses during the *decisional epoch* are steeper in trials where the Standard (averted) occurs 2^nd^, (Fig. 3a, right panel); a main effect of Trial Difficulty (F(1, 9) = 25.7, p = 3^e−04^) indicating that harder trials are characterized by steeper pupil dilation responses, (Fig. 3b, right panel); and a significant Stimulus order × Trial Difficulty interaction (F(1, 9) = 97.8, p = 4^e−06^), indicating that the dissociation in pupil response between hard and easy trials is only observable when the decisional phase directly follows the Probe, (Fig. 3c, right panel).

## Discussion

In this study, we showed that pupil dilation hazard rates predict the perceived duration of sub-second gaze shifts. We observed that the pupil dilates at a relatively linear rate from the onset of the timed interval, suggesting an accumulation of evidence that triggers a timing decision once a threshold is met. Classifications of ‘longer’ duration were associated with a faster rate of pupil dilation. This can be conceptualized in terms of arousal signals providing integrated ‘clock pulses’ for estimating the duration of gaze shifts: i.e. arousal provides a ‘clock signal’. Since arousal ramps up, greater arousal is indicative of a longer interval. Arousal, as is the case of most biological signals, is however subject to spontaneous fluctuations. Therefore, if greater arousal is achieved in the same physical time interval, that interval will be judged as longer. Differences in the rate of pupil dilation were not seen during duration classifications of equivalent Gabor carrier phase shifts, suggesting that arousal signals are only available, and therefore can be used as a clock signal, in the case of the timing of gaze behaviours.

We also observed that pupil responses predicted duration judgements only in the 2^nd^ interval (Direct 2^nd^ or Averted 2^nd^). This suggests that in conditions of maximum uncertainty, participants passively relied on the 1^st^ stimulus as a reminder of the target duration, while they actively encoded the duration of the 2^nd^ stimulus to perform the classification task. We also found differences in pupil responses within the decisional phase following the stimulus presentation. Pupil responses during the decisional phase are steeper in trials where the Standard (averted) occurs second. This mirrors previous findings relating stimulus order in Standard/Probe comparison tasks to discrimination thresholds^16^: trials in which Standard is second have higher discrimination thresholds and are therefore more effortful. We also found that pupil responses differed between hard and easy trials only when the Standard (Averted) comes first and the decisional phase follows the Probe (Direct). This suggests that, when presented first, the Standard acts as a reminder of the target duration, the Probe is then encoded and a decision process follows the Probe’s offset. If the Probe comes first and is either very short or long (Easy trials), the discrimination can be easily performed prior to the onset of the Standard, and the ensuing decisional epoch does not reveal a decision-making process.

These findings are consistent with developments in the time perception literature, in which the idea of a “central-clock”^17^ has given way to modality and task dependent systems, as well as systems specific for shorter (<1 sec) or longer (>1 sec) time scales^10, 18, 19^. Electrophysiological studies have identified various cortical endogenous signals which form a basis for timekeeping^11, 20, 21^ and temporal decision making strategies^22, 23^. While arousal has been widely known to affect timing^15^, here we identify arousal as a candidate endogenous timing signal. We demonstrate for the first time, through a data driven reverse correlation approach, that different rates in arousal directly lead to different perceived gaze times, thus showing that arousal provides a timing signal for the encoding and classification of gaze shift duration.

## Methods

### Participants

We recruited 20 participants with normal or corrected to normal vision (Experiment 1: 7 female & 3 male, 27.1+/−3.4 years; Experiment 2: 3 Female & 7 Male, 33.6+/−12 years). We chose n = 10 per experiment based on comparable sample sizes in similar pupillometry studies^24, 25^. All participants had normal or corrected to normal vision. Informed consent was obtained from all participants prior to starting the experiment. The study was approved by the UCL Research Ethics committee and was in agreement with the UCL research guidelines and regulations.

### Experimental task

Participants compared durations of gaze shifts sequentially performed by two avatar face stimuli (Fig. 1a). At the beginning of each trial, the eyes of both avatars faced away from the screen centre (i.e. left avatar started with a leftward averted gaze; right avatar started with a rightward averted gaze) and participants were required to fixate on a point positioned over the left avatar’s nasion region. After a brief pause between 1 and 2 s (randomly selected from a uniform distribution), the left avatar performed a transient rightward gaze shift. Participants were instructed to hold fixation on the left face until the avatar reset to its starting leftward gaze position. After this, participants were required to saccade to the right fixation point (positioned over the right avatar nasion region), wait for the right avatar to perform a transient leftward gaze shift (after a brief pause between 1 and 2 s) and hold fixation until they responded (indicate with a button press whether the 1^st^ or 2^nd^ gaze shift lasted longer) at the end of the trial. After responding, participants fixated on the left avatar face and waited for the next trial to start. On each trial one avatar performed an averted shift (which provided a Standard duration) while the other avatar performed a direct shift (which provided a Probe duration). The order of direct and averted shifts was counterbalanced across trials, so on half the trials the left avatar performed an averted shift and then the right avatar performed a direct shift, while in the remaining half the left avatar performed a direct shift and then the right avatar performed an averted shift (Fig. 1a). A practice block, in which transient changes in colour of the fixation points cued the relevant stimulus, ensured that participants could successfully conform to the stimulus procedure. Fixation colour was maintained constant (black) throughout experimental blocks. Both avatars remained on the screen throughout the whole experiment, thus ensuring a constant screen luminance. We tested sub-second (617 ms) and supra-second (1233 ms) Standard durations in separate blocks. Probe durations varied in 7 exponentially spaced steps: 233, 322, 446, 617, 853, 1180 and 1633 ms (in the sub-second block) and 466, 645, 892, 1233, 1707, 2361 and 3266 ms (in the supra-second block). We collected 40 repetitions per data point. Avatar stimuli were created with Poser 9 (SmithMicro Software, http://my.smithmicro.com/poser-3d-animation-software.html).

In Experiment 2, participants compared the duration of Standard (sub and supra-second) and Probe Gabor carrier phase shifts (Supplementary Information). Both stimuli sequentially performed an inward ¼ cycle phase shift. Aside for the stimuli, all other experimental features were identical to Experiment 1.

### Behavioural data analysis

We recoded “1^st^ OR 2^nd^ gaze shift longer” responses in terms of whether the Direct shift was judged Longer OR Shorter than the Averted shift. We fit participants’ proportions of “Direct shift longer” responses as a function of Direct shift duration (expressed in log space) with a cumulative Gaussian. The 50% point of this function yielded an estimate of the participant’s Point of Subjective Equality (PSE), i.e. the duration required for a Direct gaze shift to appear equal in duration to an Averted gaze shift.

### Eyetracking

Eyetracking was performed using an EyeLink1000 (http://www.sr-research.com/), sampling eye position and pupil diameter at 60 Hz (constrained by monitor refresh rate). Eye data was monitored in real time throughout each trial: trials were repeated when eye signal was lost for more than 200 ms, or when eye position deviated more than 50 px (approx. 1.2 cm) from the currently attended fixation point. Eye position was calibrated at the beginning of the gaze task with a custom-built algorithm evaluating fixations on a 3 × 3 dot array (encompassing 520 vertical × 520 horizontal pixel area). Drift correction was performed every 10 trials on a single central dot. Position and pupil data were further processed through a custom-built filtering algorithm that substituted signal losses with position/pupil data interpolated from data recorded prior and following the loss of signal.

### Pupil signal recording and processing

We recorded pupil diameter, a proxy of cortical arousal^26^, and, identified within each trial, epochs for the encoding of Direct and Averted gaze-shifts of equal duration (both either 617 ms or 1233 ms), and epochs for the Decisional phase leading to the participant’s response (Fig. 1c). We averaged pupil data within Direct and Averted encoding epochs across trials based on participants’ classification of gaze duration, yielding an averaged pupil signal for Direct classified as longer, Averted classified as longer, Direct classified as shorter and Averted classified as shorter. We ran a Principal Component Analysis (PCA) on pupil signals and tested differences in participants’ 1^st^ component score (PC1 score, higher score = greater increase in pupil diameter; see Supplementary Information).

## Electronic supplementary material

Supplementary Information
