# Supplementary material for: Pupil response hazard rates predict perceived gaze durations
Source: Sci Rep. 2017 Jun 21;7:3969. doi: 10.1038/s41598-017-04249-9 (PMC5479779; doi:10.1038/s41598-017-04249-9)
Supplement: Supplementary file 1 — Supplementary Information [file 41598_2017_4249_MOESM1_ESM.pdf]

Pupil response hazard rates predict perceived gaze durations

Nicola Binetti[1], Charlotte Harrison[1], Isabelle Mareschal[2] & Alan Johnston[1,3,4]

[1]Department of Experimental Psychology, University College London, UK

[2]School of Biological and Chemical Sciences, Psychology, Queen Mary University of London, UK

[3]CoMPLEX, University College London, UK

[4]School of Psychology, University of Nottingham, UK

Correspondence: nicolabinetti@gmai.com

Supplementary information

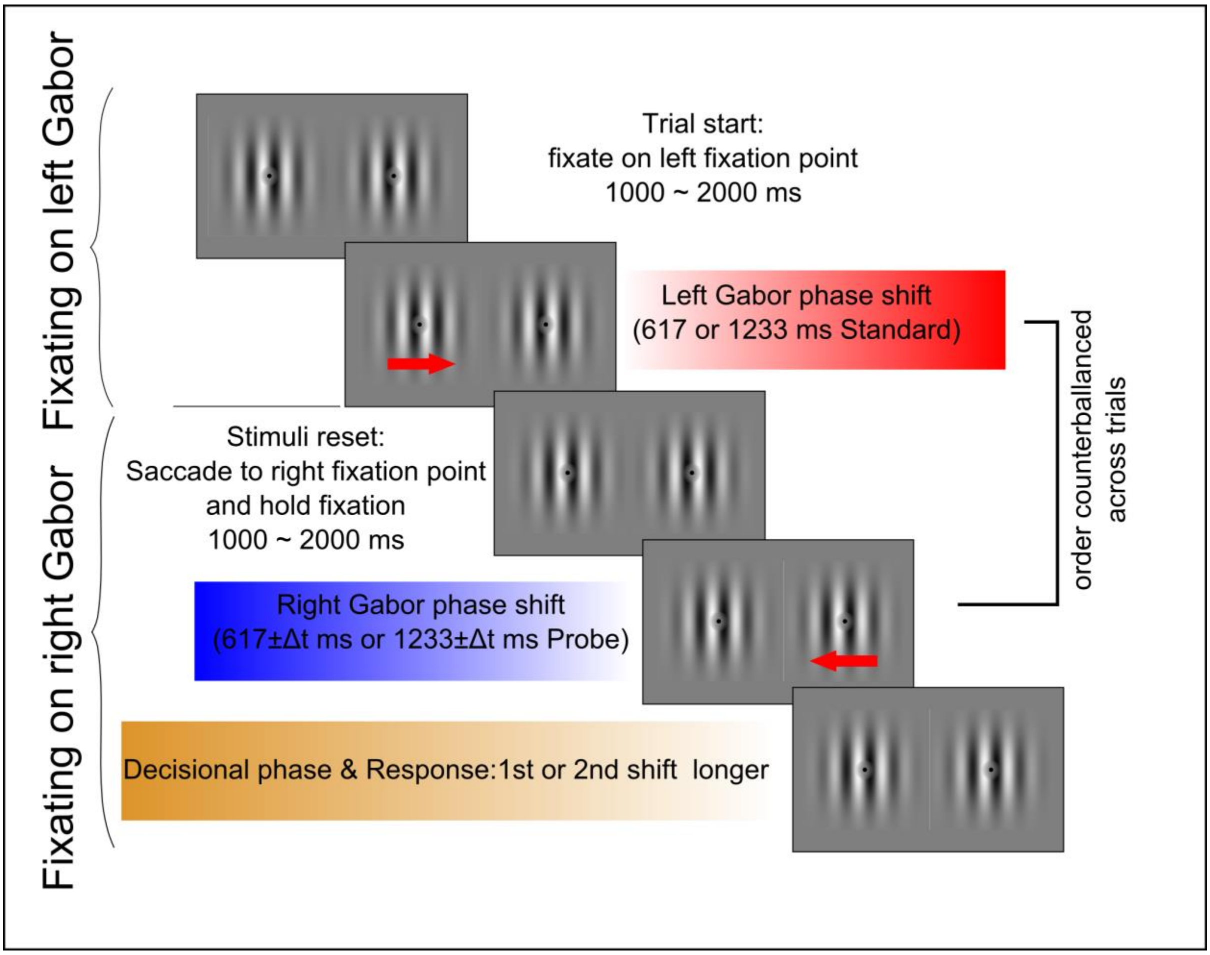

Figure S1: Gabor shift timing experimental procedure. The left and right Gabors sequentially performed an inward ¼ cycle phase shift. Participants indicated which one of the spatial shifts lasted longer.

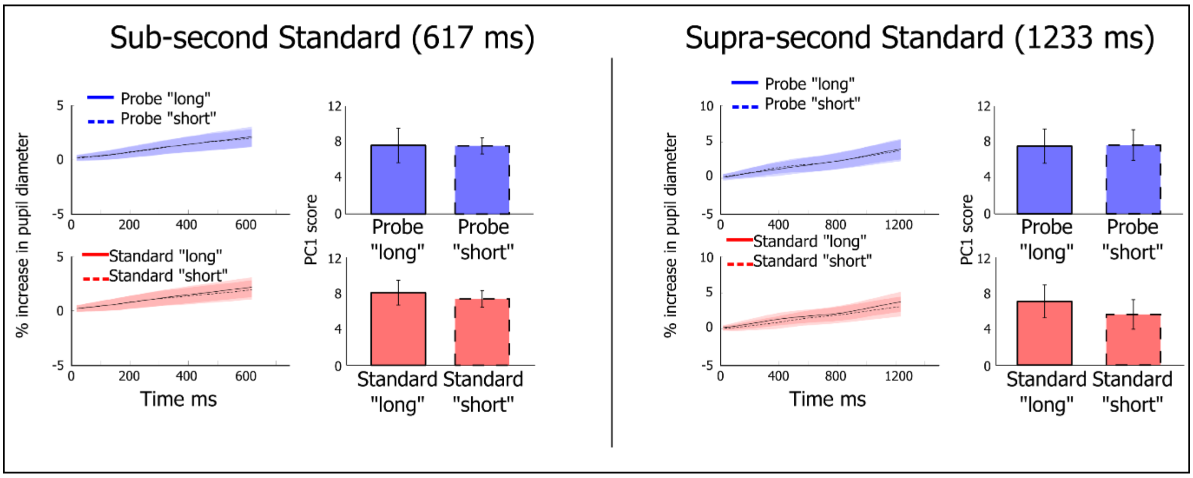

Figure S2: Averaged pupil responses during the *duration encoding* epochs of Gabor phase shifts (Experiment 2) as a function of duration judgement (longer or shorter), for sub and supra-second intervals.

Experimental setup and task

At the beginning of each trial participants were required to fixate on the left fixation point, positioned on the left avatar's nasion region. Participants were instructed to hold this fixation until the left avatar reset to its starting position following a transient rightward gaze shift (averted on 50% of trials). After this participants would saccade to the right fixation point (right avatar nasion), wait for the right avatar gaze shift and hold fixation until they responded at the end of the trial. The task was performed in a dimly lit testing environment. Stimuli were presented on a Mitsubishi Diamond Plus 250SB CRT monitor (1280x1024 pixels running at 60Hz) at a 57cm viewing distance. Head position was restrained by a headrest. Stimuli presentation and response collection were implemented on MATLAB 2013 (The Mathworks), with the Psychtoolbox 3 library running on a DELL precision T3500.

Behavioural data analysis and results

We fit participants' proportion of "Direct shift longer" responses as a function of Direct shift duration with a cumulative Gaussian. The 50% point of this function yielded an estimate of the participant's Point of Subjective Equality (PSE), i.e. the duration required for a Direct gaze shift to appear equal in duration to an Averted gaze shift. A t-test comparison revealed that gaze timing did

not significantly differ between Direct and Averted gaze shifts (Short stimuli: $t(9)=-1.88$, $p=.26$; Long stimuli: $t(9)=-.1$, $p=.34$).

Pupil signal recording, analysis and results

Eye data recording

Eyetracking was performed on an EyeLink1000 (http://www.sr-research.com/) kit, sampling eye position and pupil diameter at 60Hz. Eye data was monitored in real time throughout each trial: trials were repeated when eye signal was lost for more than 200ms, or when eye position deviated more than 50px (approx. 1.2cm) from the currently attended fixation point.

Eye data calibration and filtering

Eye position was calibrated at the beginning of the gaze task with a custom algorithm evaluating fixations on a 3x3 dot array (encompassing 520 vertical x 520 horizontal pixel area). Drift correction was performed every 10 trials on a single central dot. Position and pupil data were further processed through a custom filtering algorithm that substituted signal losses with position / pupil data interpolated from data recorded prior and following the loss of signal.

Pupil Foreshortening Effect correction

Since our task involved two fixation points, we had to compensate for apparent changes in pupil diameter due to variations in the relative angle between the eye and the camera: i.e. compensate for pupil foreshortening as the eye rotates away from the camera [1,2]. We implemented a PFE correction technique by Hayes & Petrov [3], based on a geometric model that expresses the foreshortening of the pupil area as a function of the cosine of the angle between the eye-to-camera axis and the eye-to-stimulus axis.

Pupil dilation analysis

For each epoch (Direct shift encoding, Averted shift encoding and Decisional) we expressed pupil signal as a percentage increase in pupil diameter with respect to a 200ms baseline preceding the onset of each epoch. In order to quantify changes in pupil diameter, we adopted a Principal Component Analysis (PCA) approach that reduced the dimensions of pupillary response by identifying a subset of factors along the time axis which accounted for unique variance in the data [4,5]. A PCA was run on % increases in pupil diameter, with participants as observations and time samples as variables. Based on the elbow criterion [5,6], we retained the 1st component (PC1), which depicted roughly linear increases in pupil diameter as a function of time, since this explained most of the information in the original pupil signal (>91% explained variance). Finally we tested differences in participants' PC1 score (participant coordinates in PC1 space) within the duration encoding epochs (according to *Response* and *Stimulus* type grouping criterion) and within the decisional epochs (according to a task difficulty grouping criterion).

References

1 Jay, B. *Vision Res.* **1**, 418-424 (1962).

2 Spring, K. & Stiles, W. *The British journal of ophthalmology* **32**, 347 (1948).

3 Hayes, T. R. & Petrov, A. A. *Behavior research methods*, 1-18 (2015).

4 Kuchinke, L., Võ, M. L.-H., Hofmann, M. & Jacobs, A. M. *Int. J. Psychophysiol.* **65**, 132-140 (2007).

5 Lemercier, A. *et al. The Quantitative Methods for Psychology* **10**, 179-199 (2014).

6 Shlens, J. *arXiv preprint arXiv:1404.1100* (2014).
